# BAG3 regulates stability of IL-8 mRNA via interplay between HuR and miR-4312 in PDACs

**DOI:** 10.1038/s41419-018-0874-5

**Published:** 2018-08-28

**Authors:** Chao Li, Jing-Yi Jiang, Jia-Mei Wang, Jia Sun, Ming-Xin An, Si Li, Jing Yan, Hua-Qin Wang

**Affiliations:** 1 Department of Biochemistry & Molecular Biology, China Medical University, Shenyang, 110001 China; 2 Key Laboratory of Cell Biology, Ministry of Public Health, and Key Laboratory of Medical Cell Biology, Ministry of Education, China Medical University, Shenyang, 110001 China

## Abstract

Bcl-2 associated athanogene 3 (BAG3) is highly expressed in pancreatic ductal adenocarcinoma (PDAC), and its high expression appears to be a poor prognostic factor for patients with PDAC. In this study, we show that BAG3 knockdown significantly decreases migration and invasion of PDACs via reduction of interleukine-8 (IL-8) production. BAG3 knockdown regulates IL-8 expression at the posttranscriptional levels via interplay between recruitment of RNA-binding protein HuR and miR-4312. HuR binds to the cis-elements located in the 3′-untranslational region (UTR) of the IL-8 transcript to stabilize it, whereas miR-4312-containing miRNA-induced silencing complex (miRISC) is recruited to the adjacent seed element to destabilize it. The binding of HuR prevents the recruitment of Argonaute (Ago2), overriding miR-4312-mediated translation inhibition of IL-8. BAG3 knockdown decreases cytoplasmic distribution of HuR via increasing its phosphorylation at Ser202, therefore compromising its recruitment while promoting recruitment of miR-4312 containing miRISC to IL-8 transcript. Furthermore, our data indicate that only phosphorylated Ago2 at Ser387 interacts with IL-8 transcript. BAG3 knockdown increases phosphorylation of Ago2 at Ser387, thereby further promoting loading of miR-4312 containing miRISC to IL-8 transcript. Taken together, we propose that BAG3 promotes invasion by stabilizing IL-8 transcript via HuR recruitment, and subsequently suppressing the loading of miR-4312 containing miRISC in PDACs. Our results reveal a novel pathway linking BAG3 expression to enhanced PDAC metastasis, thus making BAG3 a potential target for intervention in pancreatic cancer.

## Introduction

Pancreatic ductal adenocarcinoma (PDAC) is the most common and aggressive type of pancreatic cancer. It is one of the leading causes of cancer-related mortality worldwide^1^. The primary reason for its extremely dismal prognosis is ascribed to the ability of PDAC to metastasize in early stages^2^. Thus, it is important to fully elucidate the underlying mechanisms that implicated in PDAC invasion and metastasis for development of novel therapeutic strategies.

Bcl-2 associated athanogene 3 (BAG3) is a member of cochaperone BAG family^3^. BAG3 expression is inducible by multiple stressful stimuli in many other cell types. BAG3 is expressed in many cancers and correlated with the poor prognosis of some cancers, including pancreatic^4–12^. In addition, we recently have reported that BAG3 regulates aerobic glycolysis and proliferation of PDAC via direct interaction with hexokinase 2 mRNA^13^. Nevertheless, the oncogenic potential of BAG3 are not yet fully elucidated.

Interleukine-8 (IL-8) is associated with cell proliferation, migration, and invasion in cancer by activating C-X-C motif chemokine receptor 1 (CXCR1) and CXCR2, two cell surface G-protein coupled receptors. Clinical studies have shown that IL-8 is consistently highly expressed in PDAC patients and its upregulation in pancreatic cancers is associated with increased metastatic potential and overall dismal prognosis^14–18^. Accumulating the molecular events occurring at posttranscriptional levels have a substantial impact on regulation of gene expression for tumor growth. The mRNA stability of a specific gene is modulated by many factors, including RNA-binding proteins (RBPs) and miRNAs. 3′-untranslational region (3′UTR) of IL-8 transcript contains adenylate-uridylate-rich elements (AREs), which mediates its stabilization via recruitment of ARE-binding proteins. Human antigen R (HuR) correlates with malignancy and belongs to the Hu/ELAV family of RBPs, which interacts with AU- and U-rich elements mostly present in the 3′-UTR of target mRNAs including IL-8 to regulate stability and/or translation^19–28^.

This study demonstrated that BAG3 knockdown destabilizes IL-8 transcript by hindering cytoplasmic translocation of HuR, promoting loading of miR-4312 containing miRNA-induced silencing complex (miRISC) to destabilize IL-8 transcript. The current study provides connecting evidence between BAG3 and IL-8 mRNA stability, thereby revealing a novel pathway linking BAG3 expression to enhanced PDAC metastasis.

## Materials and methods

### Culture of PDAC cell lines

BxPC3 and SW1990 cell lines were maintained in Dulbecco's Modified Eagle Medium (Sigma-Aldrich, Saint Louis, MO) and supplemented with 10% fetal bovine serum (ExCell Biology Inc., Shanghai, China).

### Knockdown of BAG3 by CRISPR/Cas9

A dual single guide RNA approach was used to knockdown BAG3 by CRISPR/Cas9 system as previously reported^18^.

### Analysis of cytokines and chemokines in PDACs culture supernatants

A panel of 40 cytokines and chemokines was analyzed in supernatants from PDAC cultures (day 5 of culture when 70% confluent) using commercially available antibody array. Supernatants from PDAC cultures were also validated for the presence of IL-6 using commercial ELISA (R&D Systems, Inc.). Samples were run in triplicate per manufacturer’s recommendations.

### IL-8-neutralization assay

To block the effects of IL-8, 5 μg/ml of anti-IL-8 antibody (R&D Systems) were added to PDAC.

### Determination of mRNA half-life

To measure the half-life of endogenous IL-8 mRNA, actinomycin D or Amanitin was added into the cell culture medium and total RNA was prepared at the times indicated and subjected to qRT-PCR analysis using specific primers. mRNA levels were normalized to 18 S rRNA and plotted as a percentage of the value at time zero (set at 100%).

### RNA immunoprecipitation (RIP)

Magna RIP^TM^ RNA-binding protein immunoprecipitation kit (Millipore) was used for RIP procedures according to the manufacturer’s protocol. Ago2 or HuR antibody was used to pull down IL-8 mRNA. After the antibody was recovered by protein A/G beads, standard qRT-PCR was performed to detect IL-8 mRNA in the precipitates.

### Detection of mature miR-4312 and miR-4436-5p

Total miRNA was extracted using High Pure miRNA Isolation Kit (Sigma-Aldrich, Shanghai, China). Reverse transcription of miRNAs and subsequent detection of miR-4312 and miR-4436-5p was performed using All-in-OneTM miRNA qRT-PCR Detection Kit (GeneCopoeia, Rockville, MD). Upstream miRNA-4312 and miR-4436-5p primers were generated by GENECHEM (Shanghai, China), and downstream universal primers were provided by detection kit. U6 was used as internal reference for semi-quantitative analysis of miR-4312 and miR-4436-5p contents.

### Generation of reporter vectors and dual-luciferase reporter assay

The 1264 bp full-length (FL) 3′-UTR of IL-8 was generated by PCR and inserted into the pMIR-REPORT^TM^ Luciferase vector (Promega, Madison, WI) just after the stop codon. The constructs containing mutants for miR-4312 sites were generated using the pMIR-REPORT^TM^ construct containing FL 3′-UTR of IL-8 by a PCR-based method as recommended in the QuickChange site-directed mutagenesis kit (Stratagene, La Jolla, CA). PDAC cells infected with lentivirus containing scramble or shRNA specific against BAG3 (shBAG3) were co-transfected with the indicated firefly luciferase reporter vector with a Renilla luciferase vector. Transfection was performed using Lipofectamine 2000 according to the manufacturer’s instructions (Invitrogen, Carlsbad, CA). After 48 h, firefly and Renilla luciferase activities were consecutively measured using the Dual-Luciferase assay as recommended by the manufacturer (Promega, Madison, WI). The firefly luciferase signals were normalized to the Renilla luciferase signal for each individual analysis, yielding relative luciferase activities. Three independent experiments were performed with triplication and the mean ± SD calculated from one representative experiment was presented.

### Label and capture nascent RNA

Newly synthesized RNA was labeled and isolated using Click-iT Nascent RNA Capture Kit (Invitrogen) as previously reported^29^. In brief, nascent RNAs were labeled with 0.2 mM of 5-ethymyl uridine, followed by biotinylation and isolation using streptavidin magnetic beads.

### Western blot analysis

Total cellular proteins were extracted using lysis buffer containing 20 mM Tris-HCl, 150 mM NaCl, 2 mM ethylenediaminetetraacetic acid, 1% Triton-X100 and protease inhibitor cocktail (Sigma-Aldrich, Saint Louis, MO). Extracted proteins were quantified using the bicinchoninic acid protein assay kit. A total of 30 μg proteins were separated using 12% sodium dodecyl sulfate polyacrylamide gel electrophoresis and transferred to polyvinylidene difluoride membrane (Millipore Corporation, Billerica, MA).

### Statistics

The statistical significance of the difference was analyzed by analysis of variance and post hoc Dunnett’s test. Statistical significance was defined as *P* < 0.05. All experiments were repeated three times, and data were expressed as the mean ± SD (standard deviation) from a representative experiment.

## Results

### Knockdown of BAG3 inhibits migration and invasion of PDACs via decreasing IL-8 production

**Fig. 1:**
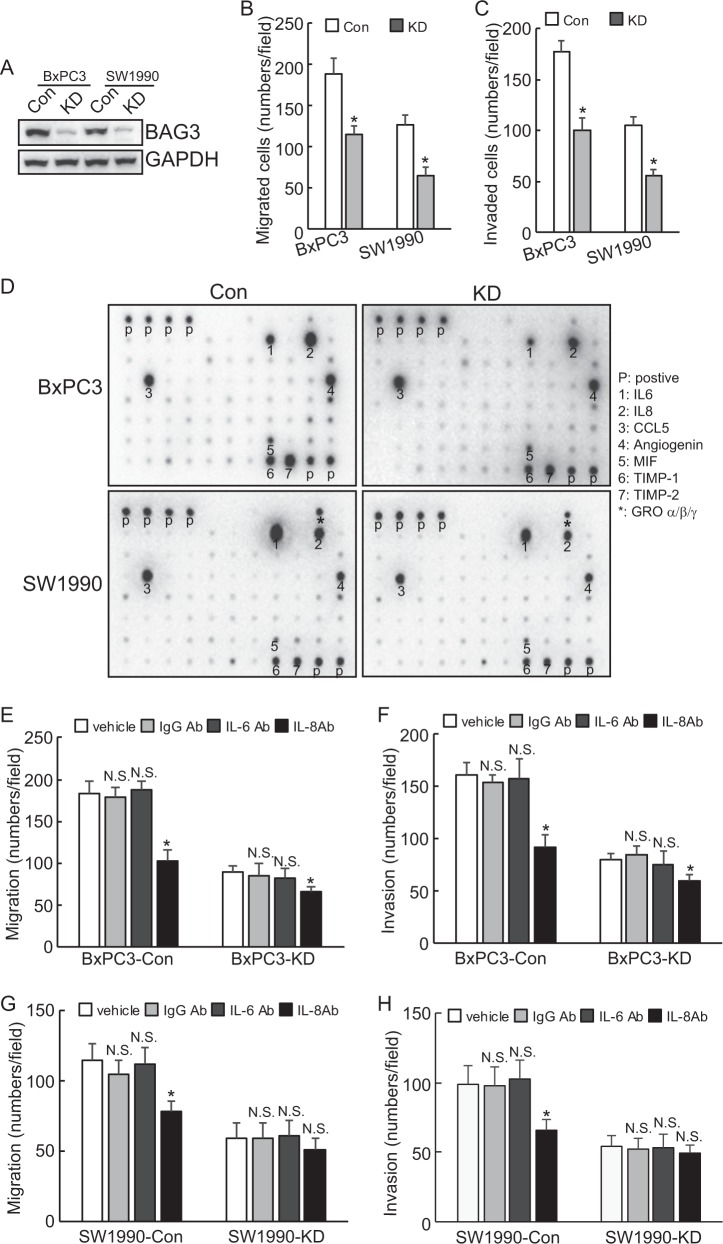
Knockdown of BAG3 inhibits migration and invasion of PDACs via reduction of IL-8 production. **a** BAG3 was knocked down using CAS9 system, and BAG3 expression was confirmed by western blot. **b**–**c** the migration and invasion of control or BAG3 knockdown (KD) cells was evaluated by a Matrigel-uncoated (**b**) and coated (**c**) transwell assay, respectively. **d** Cytokine release by control or BAG3 knockdown PDACs was analyzed using cytokine antibody microarray. **e**–**f** BxPC3 cells were exposed to the indicated antibodies, cell migration and invasion was evaluated by a Matrigel-uncoated (**e**) and coated (**f**) transwell assay, respectively. **g**–**h** SW1990 cells were exposed to the indicated antibodies, cell migration, and invasion was evaluated by a Matrigel-uncoated (**g**) and coated (**h**) transwell assay, respectively. N.S., not significant; *, *P* < 0.01

Immunoblots showed that BAG3 expression was downregulated by 80–90% in BAG3 knockdown cells (Fig. 1a). Downregulation of BAG3 significantly deceased migration (Fig. 1b) and invasion (Fig. 1c) of PDACs in transwell assays. Both cell migratory and invasive (Supplementary Figure 1A–B) capacity of PDACs with BAG3 knockdown were rescued by addition of conditional medium (CM) derived from control Cytokines antibody microarrays demonstrated that IL-6, IL-8, PDGF-BB release was decreased, whereas ICAM-1 release was increased in BxPC3 and SW1990 cells with BAG3 knockdown, and BAG3 knockdown also decreased secretion of TNFγ and TNFβ, whereas increased release of TNFα and TNF RII in SW1990 cells (Fig. 1d). As IL-8 is reported to promote invasion of PDACs, neutral antibody was used to neutralizing IL-8 in culture supernatant. Migratory and invasive capacity of BxPC3 cells were significantly decreased. The suppressive extent was more prominent in control BxPC3 cells than BAG3 knockdown (Fig. 1e, f). Although migration and invasion of BxPC3 cells was unaltered by control IgG or IL-6 antibody (Fig. 1e, f). IL-8 antibody also significantly decreased migration and invasion of control SW1990 cells (Fig. 1g, h). On the contrast, migration and invasion of SW1990 cells with BAG3 knockdown was unaltered by IL-8 antibody (Fig. 1g, h). In addition, CM derived from parental control cells mediated promotion of migration and invasion of PDACs with BAG3 knockdown was significantly suppressed by addition of IL-8 antibody (Supplementary Figure 1C–D).

### BAG3 knockdown destabilizes IL-8 mRNA in PDACs via its 3′-UTR

**Fig. 2:**
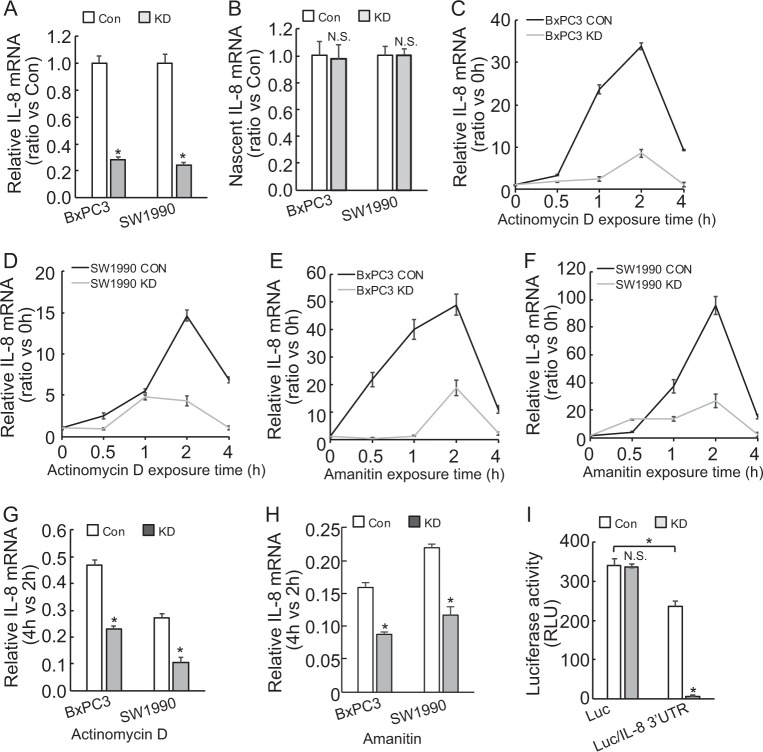
BAG3 knockdown destabilizes IL-8 mRNA via its 3′-UTR. **a** IL-8 mRNA was analyzed using real-time RT-PCR. **b** Nascent RNA was labeled and isolated, newly synthesized IL-8 mRNA was analyzed using qRT-PCR. **c**–**d** Actinomycin (**d**) was added for the indicated period to block RNA synthesis, and IL-8 mRNA was analyzed using qRT-PCR in BxPC3 (**c**) or SW1990 **d** cells. **e**–**f** Amanitin was added for the indicated period to block RNA synthesis, and IL-8 mRNA was analyzed using qRT-PCR in BxPC3 (**e**) or SW1990 (**f**) cells. **g**–**h** Relative IL-8 mRNA expression at 4 h of Actinomycin D (**g**) or Amanitin (**h**) was normalized by that of 2 h. I, BxPC3 cells were transfected with the indicated luciferase reporter vector and a Renilla reporter vector. Luciferase activity was measured 2 days after transfection and Renilla activity was measured to normalize luciferase activity. N.S., not significant; *, *P* < 0.01

To investigate the possible mechanisms underlying suppression of IL-8 release by BAG3 knockdown, Using real-time PCR demonstrated that IL-8 mRNA was significantly decreased in PDACs with BAG3 knockdown (Fig. 2a). However, de novo IL-8 mRNA synthesis was unaltered by BAG3 knockdown (Fig. 2b), indicating that BAG3 knockdown suppressed IL-8 expression at the posttranscriptional level. RNA synthesis inhibitor Actinomycin D was used to measure the half-life of IL-8 mRNA. Unexpectedly, short time exposure of PDACs to Actinomycin D resulted in significant increase in IL-8 mRNA levels (Fig. 2c, d). Another RNA synthesis inhibitor Amanitin also increased IL-8 mRNA levels within 2 h (Fig. 2e, f). As the increase reached peak at 2 h of exposure, then decreased abruptly at 4 h (Fig. 2c–f), the remained IL-8 mRNA at 4 h relative to those at 2 h was significantly decreased in PDACs with BAG3 knockdown (Fig. 2g, h). As 3′-UTR plays critical role in stability of mRNAs, fragment of 3′-UTR of *IL-8* gene was inserted after the stop codon of luciferase (Luc) gene. BAG3 knockdown significantly decreased luciferase activity of the reporter construct containing 3′-UTR of IL-8 (Fig. 2i). We insertion of IL-8 3′-UTR significantly decreased the luciferase activity (Fig. 2i), indicating that 3′-UTR of IL-8 contains unstable element(s). In addition, the degree of luciferase activity suppression (Fig. 2i) mediated by BAG3 knockdown was more apparent than IL-8 mRNA (Fig. 2a), indicating that BAG3 knockdown might also reduce the translational efficiency of IL-8 mRNA functioning as template.

### Increase in phosphorylation of HuR at Ser202 is implicated in destabilization of IL-8 mRNA by BAG3 knockdown in PDACs

**Fig. 3:**
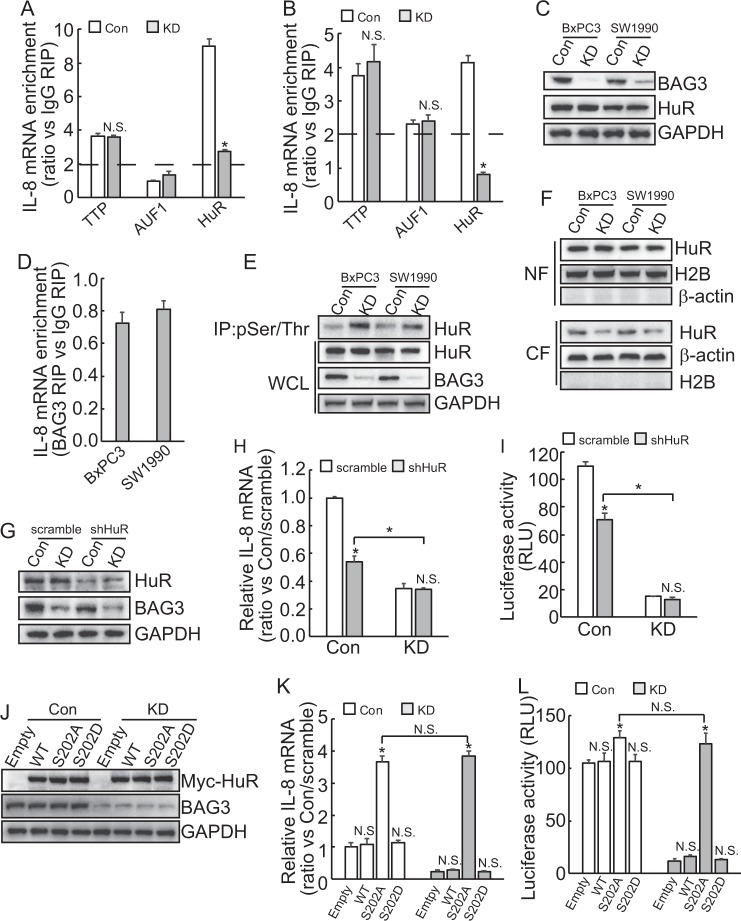
BAG3 knockdown destabilizes IL-8 mRNA via increasing HuR phosphorylation at Ser202. **a**–**b** RIP was performed using the indicated antibodies, enrichment of IL-8 mRNA was measured by qRT-PCR in BxPC3 (**a**) and SW1990 (**b**) cells. **c** HuR protein levels were measured using western blot. **d** RIP was performed using the antibody against BAG3, enrichment of IL-8 was analyzed using qRT-PCR. **e** Cell lysates were immunoprecipitated by pan-phospho Ser/Thr antibody, western blot was then performed to analyze HuR phosphorylation. **f** Cell lysates were fractioned to nuclear (NF) and cytoplasmic (CF) fraction, western blot was performed using both fractions. **g** BxPC3 cells were infected with lentivirus containing shRNA against HuR (shHuR), HuR knockdown efficiency was confirmed by western blot. **h** BxPC3 cells were infected with lentivirus containing shHuR, IL-8 mRNA expression was analyzed using qRT-PCR. **i** Luciferase containing IL-8 3′-UTR was transfected to the indicated cells, luciferase activity was analyzed. **j**–**k** Control or BAG3 KD BxPC3 cells were transfected with wild type (WT), mutation at Ser202 to alanine (S202A) or to aspartic acid (S202D) HuR, HuR expression was confirmed by western blot **j**, IL-8 mRNA was analyzed using qRT-PCR **k**. **l** BxPC3 cells were co-transfected with the indicated HuR construct and luciferase construct containing IL-8 3′-UTR, luciferase activity was analyzed. N.S., not significant; *, *P* < 0.01

Several RNA-binding proteins have been reported to be involved in the stability of IL-8 mRNA via interaction with AREs present in the 3′-UTR of IL-8 mRNA. RIP demonstrated that enrichment of IL-8 transcript by TTP or AUF1 was unaltered by BAG3 knockdown in PDACs (Fig. 3a, b). On the contrast, interaction of HuR with IL-8 mRNA was significantly suppressed by BAG3 knockdown (Fig. 3a, b). Western blot demonstrated that HuR expression was unaltered by BAG3 knockdown in PDACs (Fig. 3c). We have found that BAG3 per se interacted with some mRNAs, RIP demonstrated no interaction of BAG3 with IL-8 transcript in PDACs (Fig. 3d). Quantitative phosphoproteomics identified that phosphorylation of HuR at Ser202 was increased by BAG3 knockdown in BxPC3 cells (supplementary Figure 2A). Immunoprecipitation using antibody against pan-Ser/Thr phosphorylation confirmed that BAG3 knockdown increased HuR phosphorylation in PDACs (Fig. 3e). It has been reported that phosphorylation of HuR at Ser202 entraps it in the nucleus^30–32^. Nuclear fractionation was demonstrated that nuclear HuR was unaltered, whereas cytoplasmic HuR was significantly decreased in PDACs with BAG3 knockdown (Fig. 3f). Cytoimmunofluorescence confirmed that cytoplasmic distribution of HuR was significantly decreased in BAG3 knockdown BxPC3 and SW1990 cells (Supplementary Figure 2B–C). To investigate the potential involvement of HuR in BAG3 knockdown-mediated destabilization of IL-8 mRNA, HuR was knockdown using lentivirus containing shRNAs (shHuR) (Fig. 3g). Real-time PCR demonstrated that downregulation of HuR reduced IL-8 mRNA expression in control, whereas had no obvious effect in BAG3 knockdown cells (Fig. 3h). ShHuR significantly decreased luciferase activity of construct with insertion of IL-8 3′-UTR in control, whereas no obvious effect in BAG3 knockdown cells (Fig. 3i). Knockdown of HuR unaltered the luciferase activity of control (data not show). It should be noted that IL-8 mRNA expression (Fig. 3h), and the luciferase activity (Fig. 3i) of BxPC3 cells with BAG3 knockdown was significantly lower than control even when HuR was knockdown. HuR was also knockdown in SW1990 cells (Supplementary Figure 3A). IL-8 mRNA was decreased by HuR knockdown in control, whereas unaltered in BAG3 knockdown SW1990 cells (Supplementary Figure 3B).

To further explore the potential implication of HuR phosphorylation at Ser202, BxPC3 cells were transfected with constructs containing WT, nonphosphorylatable mutant (S202A) or phosphorylation mimetic mutant (S202D) HuR (Fig. 3j). IL-8 mRNA was increased by ectopic expression of S202A HuR, whereas unaltered by WT or S202D HuR in both control and BAG3 knockdown cells (Fig. 3k). Unexpectedly, BAG3 knockdown cells expressed comparable IL-8 mRNA with control BxPC3 cells when S202A HuR was forced expressed (Fig. 3k). Similar phenomena were observed in SW1990 cells (Supplementary Figure 3C-D). S202A HuR significantly increased the luciferase activity of construct with insertion of IL-8 3′-UTR, whereas WT or S202D HuR demonstrated no effect in both control and BAG3 knockdown BxPC3 cells (Fig. 3l). Consistent with IL-8 mRNA expression, BAG3 knockdown cells and control demonstrated similar luciferase activity of construct with insertion of IL-8 3′-UTR when S202A HuR was forced expressed (Fig. 3l). Overexpression of intact or mutant HuR unaltered the luciferase activity of control reporter construct (data not shown).

### miRISC complex is implicated in destabilization of IL-8 mRNA by BAG3 knockdown in PDACs

**Fig. 4:**
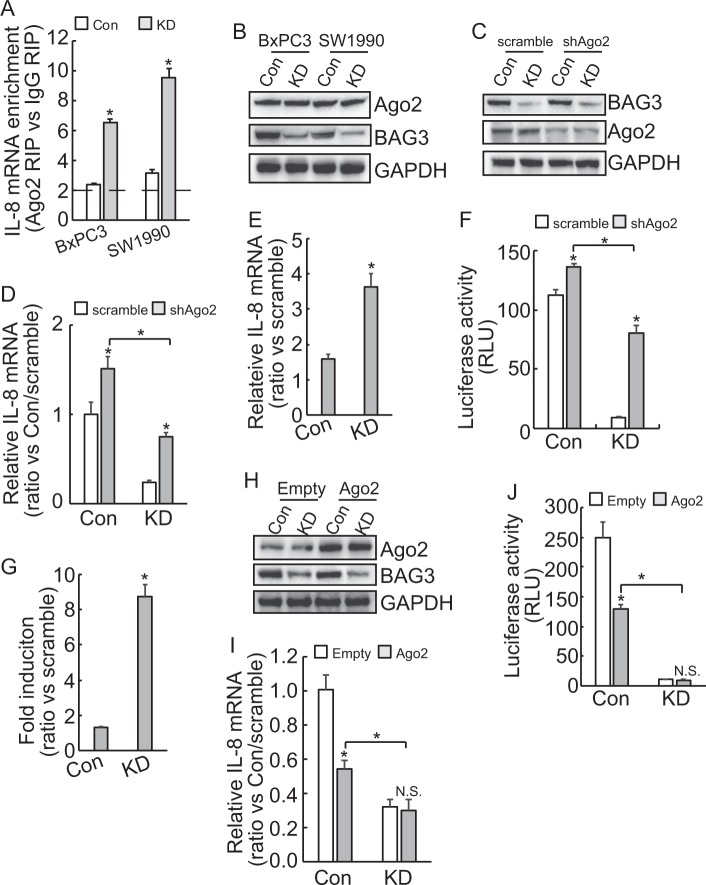
BAG3 knockdown destabilizes IL-8 mRNA via miRISC complex. **a** RIP was performed using Ago2 antibody, enrichment of IL-8 mRNA was analyzed using qRT-PCR. **b** Ago2 protein expression was analyzed using western blot. **c** BxPC3 cells were infected with lentivirus containing Ago2 (shAgo2), knockdown efficiency wax confirmed by western blot analysis. **d**–**e** BxPC3 cells were infected with lentivirus containing shAgo2, IL-8 mRNA was analyzed using qRT-PCR. **f**–**g** The indicated BxPC3 cells were transfected with luciferase containing IL-8 3′-UTR, luciferase activity was analyzed. **h**–**i** Control or BAG3 KD BxPC3 cells were transfected with Ago2, Ago2 expression was confirmed by western blot (**h**), IL-8 mRNA was analyzed using qRT-PCR **i**. **j** BxPC3 cells were co-transfected with Ago2 and luciferase construct containing IL-8 3′-UTR, luciferase activity was analyzed. N.S., not significant; *, *P* < 0.01

When HuR was downregulated, BAG3 knockdown cells exhibited reduced IL-8 mRNA (Fig. 3h) and luciferase activity (Fig. 3i), compared with control, whereas in the presence of S202A HuR, BAG3 knockdown cells and control expressed comparable IL-8 mRNA (Fig. 3k) and luciferase activity (Fig. 3l). These data indicated that additional factor(s) might be responsible for suppression of IL-8 by BAG3 knockdown. As miRISC plays critical roles in posttranscriptional regulation of target mRNAs via recruitment to 3′-UTR^33^. RIP using antibody against Ago2 was explored. More than twofold enrichment of IL-8 mRNA by Ago2 was observed in control BxPC3 and SW1990 cells, indicating that IL-8 transcript might be a target of miRISC generally. Importantly, recruitment of Ago2 to IL-8 transcript was significantly enhanced by BAG3 knockdown (Fig. 4a). Western blot demonstrated that BAG3 knockdown unaltered Ago2 expression (Fig. 4b). To explore the potential involvement of miRISCs, Ago2 was downregulated using shAgo2 in BxPC3 cells (Fig. 4c). Real-time PCR demonstrated that knockdown of Ago2 increased IL-8 mRNA in both control and BAG3 knockdown cells (Fig. 4d). The degree of IL-8 mRNA upregulation was more apparent in BAG3 knockdown cells (Fig. 4e). Knockdown of Ago2 increased the luciferase activity of reporter construct containing IL-8 3′-UTR in both control and BAG3 knockdown cells (Fig. 4f). The extent of luciferase activity increase was more apparent in BAG3 knockdown cells (Fig. 4g). BAG3 knockdown decreased IL-8 mRNA (Fig. 4d) and luciferase activity (Fig. 4f) even when Ago2 was downregulated. Knockdown of Ago2 had no effect on the luciferase activity of control reporter construct (data not shown). To further confirm the involvement of miRISCs, Ago2 was overexpressed in BxPC3 cells (Fig. 4h). Ago2 overexpression significantly decreased IL-8 mRNA and luciferase activity of reporter construct containing IL-8 3′-UTR in control, whereas had no obvious effect in BAG3 knockdown BxPC3 cells (Fig. 4I, j). When Ago2 was forced expressed, BAG3 knockdown still decreased IL-8 mRNA (Fig. 4i) and luciferase activity (Fig. 4j).

### Interplay between miRISC complex and HuR is involved in posttranscriptional regulation of IL-8 by BAG3 knockdown in PDACs

**Fig. 5:**
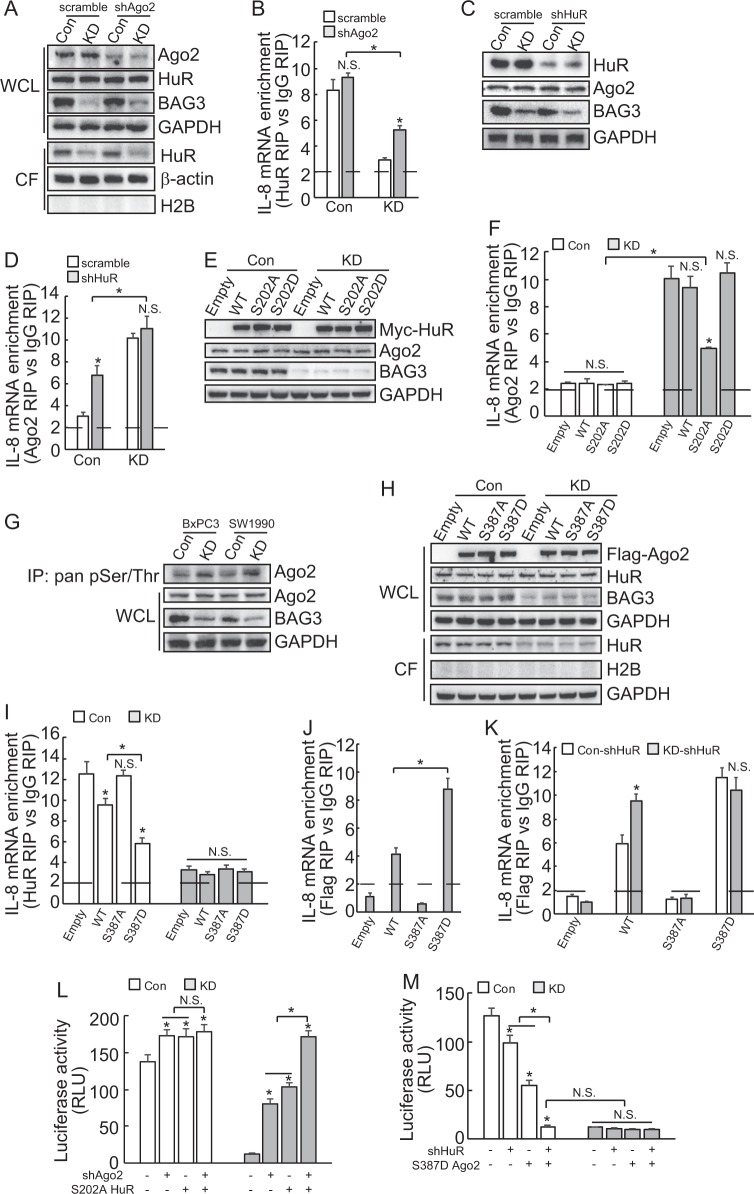
BAG3 knockdown destabilizes IL-8 mRNA via interplay between miRISC complex and HuR. **a** BxPC3 were infected with lentivirus containing shAgo2, HuR protein in whole-cell lysates (WCL) as well cytoplasmic fraction (CF) was analyzed using western blot analysis. **b** RIP was performed using HuR antibody, enrichment of IL-8 mRNA in the indicated cells was analyzed using qRT-PCR. **c**–**d** control or BAG3 KD BxPC3 cells were infected with lentivirus containing scrmble or shHuR, Ago2 expression was analyzed using western blot **c**, enrichment of IL-8 mRNA by Ago2 was analyzed using RIP followed by qRT-PCR **d**. **e**–**f** control or BAG3 KD BxPC3 cells were transfected with WT, S202A, or S202D HuR, Ago2 expression was analyzed using western blot **e** enrichment of IL-8 mRNA by Ago2 was analyzed using RIP followed by qRT-PCR **f**. **g** Immunoprecipitation was performed using pan-phospho Ser/Thr antibody, Ago2 phosphorylation was then analyzed by western blot. **h**–**i** BxPC3 cells were transfected with wild type (WT), mutation at Ser387 to alanine (S387A), or to aspartic acid (S387D) Ago2. HuR expression was analyzed in both whole-cell lysate and cytoplasmic fraction using western blot **h**, and enrichment of IL-8 mRNA by HuR was analyzed using RIP followed by qRT-PCR **i**. **j** BxPC3 cells were transfected with the indicated Ago2 construct tagged with Flag, enrichment of IL-8 mRNA by Ago2 was measured using RIP followed by qRT-PCR. **k** control or BAG3 KD cells were infected with lentivirus containing shHuR, then transfected with the indicated Ago2 construct. Enrichment of IL-8 mRNA by Ago2 was analyzed by RIP followed by qRT-PCR. **l**–**m** BxPC3 cells were co-transfected with the indicated construct and luciferase containing IL-8 3′-UTR, luciferase activity was analyzed post 48 h transfection. N.S., not significant; *, *P* < 0.01

In order to investigate whether miRISC complex and HuR might affect each other’s loading on the IL-8 mRNA, RNA immunoprecipitation using HuR and Ago2 antibodies were performed using cells infected with shHuR or shAgo2. Western blot demonstrated that knockdown of Ago2 unaltered HuR expression, and its cytoplasmic distribution in both control and BAG3 knockdown cells (Fig. 5a). RIP demonstrated that knockdown of Ago2 had no obvious effect on loading of HuR on IL-8 mRNA in control, whereas significantly increased recruitment of HuR to the IL-8 mRNA in BAG3 knockdown BxPC3 cells (Fig. 5b). It should be noted that even when Ago2 was downregulated, less HuR was recruited to the IL-8 mRNA in cells with BAG3 knockdown (Fig. 5b). Similar results were observed in SW1990 cells (Supplementary Figure 4A-C). HuR was downregulated by shHuR. Western blot demonstrated that HuR knockdown unaltered Ago2 expression (Fig. 5c). RIP demonstrated that knockdown of HuR significantly promoted recruitment of Ago2 to the IL-8 transcript in control, whereas unaltered in BAG3 knockdown BxPC3 cells (Fig. 5d). It should be noted that extent of Ago2 recruitment was less in control than that in BAG3 knockdown cells even when HuR was knockdown (Fig. 5d). Downregulation of HuR unaltered Ago2 expression (Supplementary Figure 4D), whereas significantly increased its recruitment to IL-8 mRNA in control, and knockdown HuR unaltered recruitment of Ago2 in BAG3 knockdown SW1990 cells (Supplementary Figure 4E). To further confirm the interplay between miRISC complex and HuR, HuR was overexpressed in control and BxPC3 with BAG3 knockdown cells and western blot demonstrated that neither intact nor mutant HuR altered Ago2 expression (Fig. 5e). RIP demonstrated that ectopic expression of either intact or mutant HuR unaltered the recruitment of Ago2 to the IL-8 mRNA in control BxPC3 cells (Fig. 5f). S202A mutant HuR significantly suppressed Ago2 loading on the IL-8 mRNA, whereas Ago2 recruitment was unaltered by WT or S202D mutant HuR in BAG3 knockdown BxPC3 cells (Fig. 5f). It should be noted that more Ago2 was recruited to IL-8 transcript in BAG3 knockdown cells even S202A HuR overexpressed (Fig. 5f). Quantitative phosphoproteomics identified that phosphorylation of Ago2 at Ser387 was also increased by BAG3 knockdown in BxPC3 cells (Supplementary Figure 4F). Immunoprecipitation using an antibody against pan-Ser/Thr phosphorylation confirmed that BAG3 knockdown increased Ago2 phosphorylation in PDACs (Fig. 5g). BxPC3 cells were transfected with constructs containing WT, nonphosphorylatable mutant (S387A) or phosphorylation mimetic mutant (S387D) Ago2 (Fig. 5h). Total HuR expression or cytoplasmic distribution was unaltered by intact or mutant Ago2 in both control and BAG3 knockdown cells (Fig. 5h). RIP demonstrated that WT and S387D Ago2 expression significantly decreased recruitment of HuR to the IL-8 mRNA, whereas S387A Ago2 had no effect in control BxPC3 cells (Fig. 5i). WT or mutant Ago2 unaltered the recruitment of HuR to the IL-8 mRNA obviously in cells with BAG3 knockdown (Fig. 5i). S387D Ago2 demonstrated more potent suppressive effect on HuR recruitment compared with WT (Fig. 5i). RIP using Flag antibody demonstrated more S387D Ago2 was recruited to the IL-8 transcript than WT Ago2, whereas no S387A Ago2 (Fig. 5j). RIP demonstrated that no S387A Ago2 was recruited to IL-8 transcript in either control or BAG3 knockdown cells with HuR downregulation (Fig. 5k). BAG3 knockdown cells demonstrated more WT Ago2 recruitment, whereas similar extent of S387D Ago2 recruitment was observed to control, when HuR was downregulated (Fig. 5k). To further investigate the involvement of phosphorylation of HuR and Ago2 in downregulation of IL-8 mediated by BAG3 knockdown, luciferase activity assays were performed in BxPC3 cells infected with shAgo2 and S202A HuR alone or in combination. Ago2 knockdown and S202A HuR alone or in combination increased the luciferase activity of reporter construct containing IL-8 3′UTR with similar extent in control BxPC3 cells (Fig. 5l). Combination of Ago2 knockdown and S202A HuR significantly increased the luciferase activity in BAG3 knockdown cells, when compared with Ago2 knockdown or S202A HuR alone (Fig. 5l). In addition, combination of HuR knockdown and S387D Ago2 significantly decreased the luciferase activity in control BxPC3 cells, when compared with Ago2 knockdown or S387D Ago2 alone (Fig. 5m). It should be noted that combination of HuR knockdown and S387D Ago2 resulted in similar extent of reduction to BAG3 knockdown (Fig. 5m).

### miR-4312 involves in suppression of IL-8 expression by BAG3 knockdown via competition with HuR to interact with IL-8 mRNA in PDACs

**Fig. 6:**
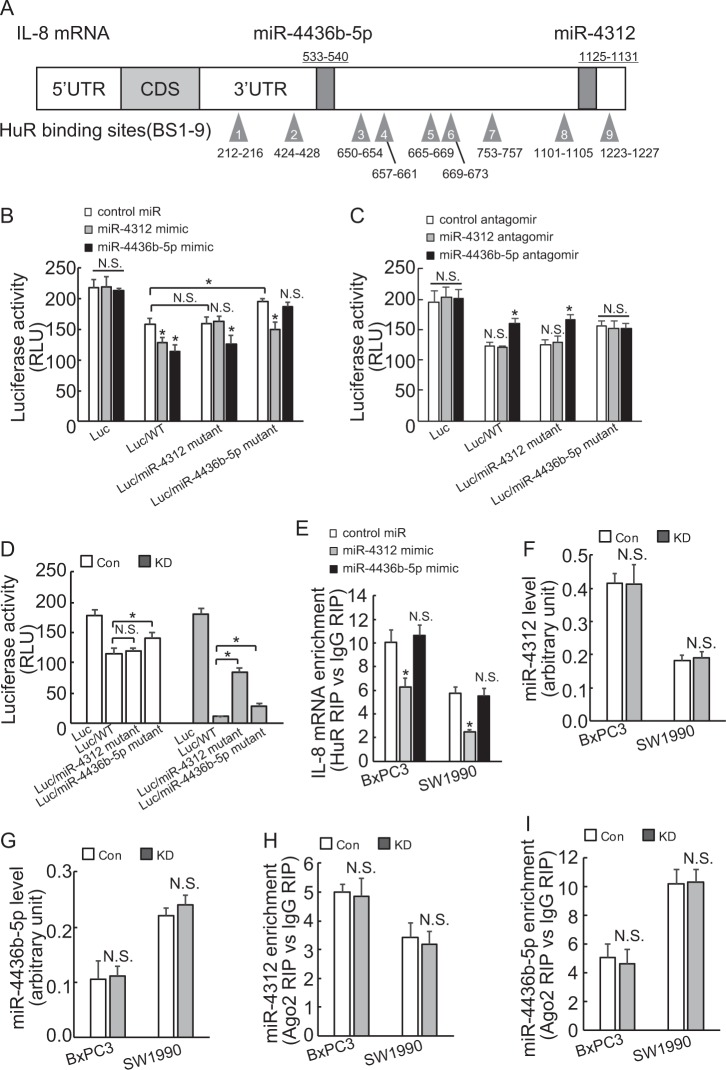
miR-4312 competes with HuR to interact with IL-8 mRNA and involves in suppression of IL-8 expression by BAG3 knockdown in PDACs. **a** Schematic illustration of HuR and miR-4436b-5p and miR-4312 potential binding sites on IL-8 mRNA. **b**–**c** BxPC3 cells were co-transfected with miR-4312 or miR-4436b-5p mimic (**b**) or antagomir (**c**) and luciferase containing IL-8 3′-UTR, luciferase activity was measured after 48 h of transfection. **d** control or BAG3 KD BxPC3 cells were transfected with luciferase construct containing wild-type (WT) or potential miRs binding site mutant IL-8 3′-UTR, luciferase activity was measured. **e** BxPC3 cells were transfected with miR-4312 or miR-4436b-5p mimic, enrichment of IL-8 mRNA by HuR was analyzed using RIP followed by qRT-PCR. **f**–**g** miR-4312 **f** and miR-4436-5p (**g**) expression levels were analyzed using qRT-PCR. **h**–**i**, miR-4312 (**h**) and miR-4436-5p (**i**) containing miRISC formation was analyzed using RIP followed by qRT-PCR. N.S., not significant; *, *P* < 0.01

The 3′-UTR of the IL-8 transcript was analyzed using miRNA databases including Targetscan and miRDB, and miR-4312 and miR-4436b-5p (Supplementary Figure 5A–B) attracted our attention, as Genechip data demonstrated they are expressed in abundance in BxPC3 cells (data not shown). In addition, both miR-4312 and miR-4436b-5p potential binding sites are adjacent or close to the binding sites of HuR on 3′-UTR of IL-8 mRNA (Fig. 6a). To evaluate the potential contribution of the miR-4312 or miR-4436b-5p-binding site in the 3′-UTR of IL-8 mRNA, intact or mutant IL-8 3′-UTR was inserted into a luciferase reporter vector just after the stop codon of *Luciferase* gene. Mutation of miR-4436b-5p potential binding site increased, whereas mutation of miR-4312 potential binding site unaltered in BxPC3 cells (Fig. 6b). Both miR-4312 and miR-4436b-5p mimics significantly reduced the reporter activity fused to the intact or irrelevant mutant IL-8 3′-UTR, whereas had no obvious effect to their respective mutant IL-8 3′-UTR (Fig. 6b). On the contrast, miR-4436b-5p antagomir significantly increased the reporter activity fused to intact or miR-4312-binding site mutant IL-8 3′-UTR, whereas no obvious effect on the reporter activity fused to its potential binding site mutant IL-8 3′-UTR in BxPC3 cells (Fig. 6c). miR-4312 antagomir unaltered the luciferase activity of any reporter construct tested in control (Fig. 6c). These data indicated that IL-8 transcript might be a target for miR-4312 and miR-4436b-5p in PDACs. BAG3 knockdown significantly decreased luciferase activity of reporter construct containing intact IL-8 3′-UTR, which was partly compromised by mutation of miR-4312 or miR-4436b-5p potential binding sites (Fig. 6d). Compared with reporter construct containing intact IL-8 3′-UTR, mutation of miR-4312-binding site unaltered the luciferase activity in control, whereas it demonstrated stronger promotive effect than that of miR-4436b-5p-binding site in BAG3 knockdown BXPC3 cells (Fig. 6d). RIP demonstrated miR-4312 mimic significantly decreased HuR recruitment to IL-8 mRNA, in contrast, miR-4436b-5p unaltered the recruitment of HuR in both BxPC3 and SW1990 cells obviously (Fig. 6e). Real-time PCR demonstrated that neither miR-4312 (Fig. 6f) nor miR-4436b-5p (Fig. 6g) expression was altered by BAG3 knockdown in PDACs. RIP demonstrated that BAG3 knockdown unaltered miR-4312 (Fig. 6h) or miR-4436b-5p (Fig. 6i) containing miRISC formation in PDACs.

### Correlation of BAG3 with IL-8 expression in pancreatic cancer specimens

**Fig. 7:**
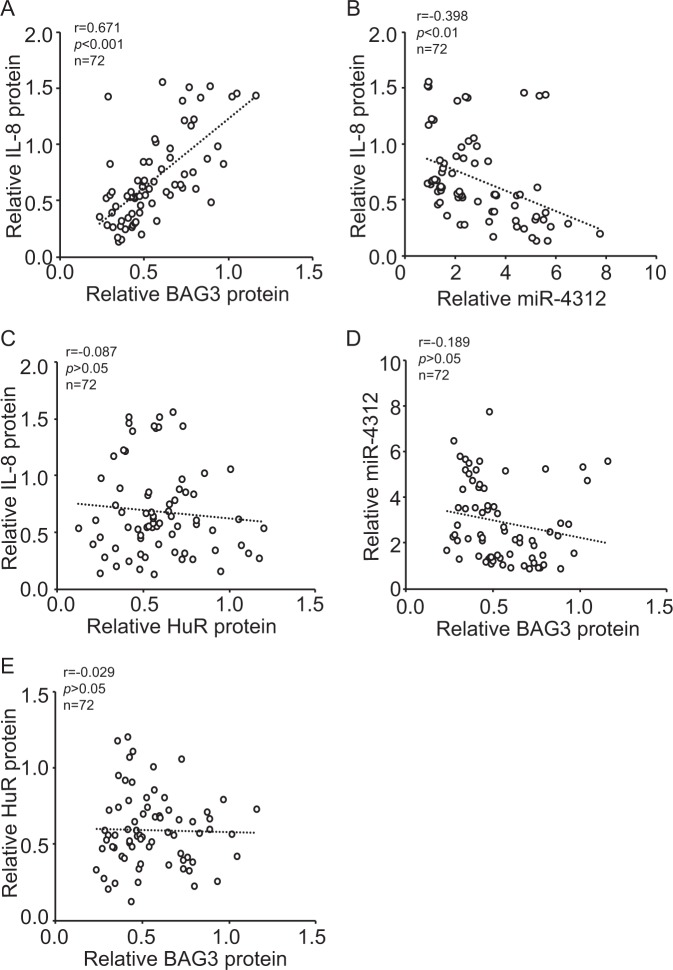
Correlation of BAG3 and IL-8 expression in pancreatic cancer tissues. **a**–**e** BAG3, IL-8, and HuR protein levels were investigated using western blot, miR-4312 levels were measured using qRT-PCR in fresh pancreatic cancer tissues. Scatter plots showing the correlation between BAG3 and IL-8 (Pearson’s coefficient test *r* = 0.671, *P* < 0.001) (**a**) miR-4312 and IL-8 (Pearson’s coefficient test *r* = −0.398, *P* < 0.01) (**b**), HuR and IL-8 (Pearson’s coefficient test *r* = − 0.087, *P* > 0.05) (**c**), as well as the correlation between BAG3 and miR-4312 (Pearson’s coefficient test *r* = −0.189, *P* > 0.05) (**d**), BAG3 and HuR (Pearson’s coefficient test *r* = −0.029, *P* > 0.05) (**e**) in pancreatic cancer tissues. Pearson’s coefficient tests were performed to assess statistical significance

Given that BAG3 might regulate IL-8 expression in PDACs, western blot were performed to evaluate the relationship between BAG3 and IL-8 in fresh pancreatic cancer specimen (Supplementary Figure 6). In pancreatic cancer tissues, BAG3 expression was positively correlated with IL-8 expression (Fig. 7a). Real-time PCR demonstrated that miR-4312 was negatively correlated with IL-8 expression (Fig. 7b). Western blot demonstrated that total HuR expression uncorrelated with IL-8 expression (Fig. 7c). Consistent with results from PDAC cell lines (Fig. 6f), no significant correlation of BAG3 and miR-4312 (Fig. 7d), or BAG3 and total HuR (Fig. 7e) was observed

**Fig. 8:**
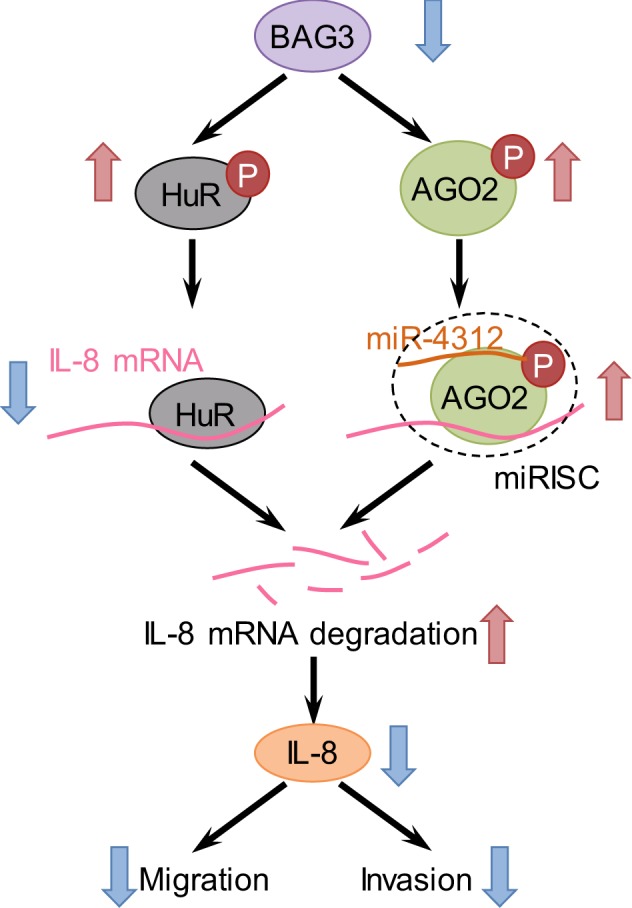
Schematic representation of destabilization of IL-8 mRNA by BAG3 knockdown in PDACs

## Discussion

BAG3 expression is constitutively expressed and regulates invasion and metastasis of some cancer^34–39^. BAG3 upregulation has been correlated with poor prognosis in patients with PDACs^13^. In this study we show that BAG3 is required for migration and invasion of PDACs via regulation of IL-8 production. IL-8 is increased and functions as an important control point in metastasis of PDACs^18^. Clinical studies have shown a high IL-8 expression correlates with an enhanced metastatic potential and overall poor prognosis in pancreatic cancers^40,41^. Posttranscriptional regulation of RNA is a powerful mediator of gene expression. Our data clearly establish that BAG3 has a key role in promoting IL-8 expression in PDACs at the posttranscriptional levels. In addition, our study uncovers a novel mechanism by which BAG3 modulates IL-8 expression via RNA-binding protein HuR and miR-4312 containing miRISC.

RNA-binding proteins and miRNAs, two main types of mRNA-interacting factors, play critical role in posttranscriptional regulation of target mRNAs via interactions with their UTR, particularly the 3′-UTR^42^. HuR is an ubiquitously expressed RNA-binding protein and considered as a definite enhancer of ARE-containing mRNA stability via binding with 3′-UTR of target mRNAs^43^. HuR plays a key role in promoting IL-8 expression by binding to its 3′-UTR at the posttranscriptional level^44–46^. HuR is predominantly distributed in the nucleus, but it translocates to the cytoplasm where it stabilizes target mRNAs and/or modulates their translation following exposure to stress and mitogens^27^. HuR is elevated in human PDAC specimens, and its cytoplasmic shift is associated with increased tumor stage and correlates with a worse prognosis^47^. Translocation of the HuR protein across the nuclear membrane is regulated by phosphorylation in a central “hinge” region of HuR. Activation of AMPK facilitates the nuclear import of HuR mediated by importin α^30^, and CDK1 phosphorylates HuR protein at Ser202, enhancing its accumulation in the nucleus^32^. In contrast, p38 MAPK^48^, PKC^49,50^, and Chk2^51^ drives the ATP-dependent export of HuR protein via phosphorylation. The current study demonstrated that BAG3 knockdown trapped HuR in the nucleus via increasing its phosphorylation at Ser202, thereby prohibiting its enrichment to IL-8 transcript.

Whereas RNA-binding proteins and miRNAs are demonstrated to regulate the stability and/or translation of specific mRNAs independently, functional interplay between specific RNA-binding proteins and miRNAs in regulating multiple genes at the posttranscriptional level has been reported recently^52^. This interplay can either be antagonistic^53^, or cooperative^54^. Accumulating evidences support that posttranscriptional regulation of a target mRNA via interplay between HuR and miRNAs play an important role in fine-tuning expression in various cellular processes, including cancer^55–66^. By linking the interplay between HuR and miR-4312 to regulation of IL-8, our data indicate a posttranscriptional mechanism that controls the expression of IL-8 by BAG3 in PDACs (Figure 8). miR-4312 site is located near HuR sites on 3′-UTR of IL-8, suggesting that HuR and miR-4312 containing miRISC might compete via their physical interaction with IL-8 mRNA possibly via steric hindrance. The competing actions of HuR and miR-4312 influenced IL-8 expression, in turn modulating invasive capacity of PDACs. Therefore, our study may open the door to new strategies to manipulate IL-8 levels in PDACs.

In this study, we have focused on the IL-8 as a molecular target of BAG3 via HuR and miR-4312. Although other mRNA targets may be affected by HuR and miR-4312, thereby contributing to the tumor phenotype, IL-8 serves as a molecular validation for our model as it is implicated as an important control point in metastasis of PDACs. These results support our hypothesis that BAG3 is important in metastasis of PDACs and the potential of this protein as a therapeutic target in pancreatic cancer. As elevated cytoplasmic HuR has been widely correlated with advanced malignancy, further studies are warranted to investigate if BAG3 regulates additional HuR target mRNAs.

## Electronic supplementary material

Supplementary Figure 1

Supplementary Figure 2

Supplementary Figure 3

Supplementary Figure 4

Supplementary Figure 5A

Supplementary Figure 5B

Supplementary Figure 6

Supplementary data
