# Supplementary material for: BAG3 regulates stability of IL-8 mRNA via interplay between HuR and miR-4312 in PDACs
Source: Cell Death Dis. 2018 Aug 28;9(9):863. doi: 10.1038/s41419-018-0874-5 (PMC6113235; doi:10.1038/s41419-018-0874-5)
Supplement: Supplementary file 8 — Supplementary data [file 41419_2018_874_MOESM8_ESM.docx]

**Supplementary data**

**Supplementary Figure Legends**

**Supplementary Figure 1 Reduction of migration and invasion of PDACs by BAG3 knockdown is rescued by conditional media from control PDACs, which is suppressed by IL-8 antibody.** A-B, BAG3 knockdown cells were cultured with conditional medium (CM) collected from control or BAG3 knockdown cells, the migration and invasion of BAG3 KD cells was evaluated by a Matrigel-uncoated (A) and coated (B) Transwell assay, respectively. C-D, CM was neutralized with the indicated antibodies, then was used to treat BAG3 knockdown cells. Cell migration and invasion was evaluated by a Matrigel-uncoated (C) and coated (D) Transwell assay, respectively. *, *P*<0.01.

**Supplementary Figure 2 BAG3 knockdown increases HuR phosphorylation at S202 site and suppresses cytoplasmic traffic of HuR in PDACs.** A. Peptide spectrum showed that phosphorylation of HuR at S202 site was increased in BxPC3 cells with BAG3 knockdown. B-C, Immunofluorescent staining of HuR in control and BAG3 knockdown BxPC3 (B) and SW1990 (C) cells.

**Supplementary Figure 3 Implication of HuR phosphorylation at S202 site in destabilization of IL-8 BAG3-mediated regulation of IL-8 expression in SW1990 cells.** A-B, Control or BAG3 knockdown SW1990 cells were infected with lentivirus containing shRNA against HuR (shHuR). HuR knockdown efficiency was confirmed by Western blot (A), and IL-8 mRNA expression was analyzed using qRT-PCR (B). C-D, Control or BAG3 KD SW1990 cells were transfected with wild type (WT), mutation at Ser202 to alanine (S202A) or to aspartic acid (S202D) HuR, HuR expression was confirmed by Western blot (C), IL-8 mRNA was analyzed using real-time RT-PCR (D).

**Supplementary Figure 4 Interplay between miRISC complex and HuR is involved in posttranscriptional regulation of IL-8 by BAG3 knockdown in SW1990 cells.** A-C, SW1990 cells were infected with lentivirus containing shAgo2, total HuR protein (A) and cytoplasmic fraction (CF) or nuclear fraction (NF) of HuR (B) was analyzed using Western blot analysis, RIP was performed using HuR antibody, enrichment of IL-8 mRNA in the indicated cells was analyzed using qRT-PCR (C). D-E, control or BAG3 KD SW1990 cells were infected with lentivirus containing scrmble or shHuR, Ago2 expression was analyzed using Western blot (D), enrichment of IL-8 mRNA by Ago2 was analyzed using RIP followed by qRT-PCR (E). F, Peptide spectrum showed that phosphorylation of Ago2 at S387 site was increased in BxPC3 cells with BAG3 knockdown.

**Supplementary Figure 5 Potential interaction between miR-4312 and miR-4436-5p is predicted by Targetscan and miRDB.**

**Supplementary Figure 6 Correlative expression of BAG3 and IL-8 in pancreatic cancer tissues.** Western blot analyses were performed using fresh pancreatic cancer tissues with the indicated antibodies and images were provided.
